# Bayesian Diagnosis of Occlusion Myocardial Infarction: A Case-Based Clinical Analysis

**DOI:** 10.3390/diagnostics15172148

**Published:** 2025-08-25

**Authors:** José Nunes de Alencar, Hans Helseth, Henrique Melo de Assis, Stephen W. Smith

**Affiliations:** 1 Instituto Dante Pazzanese de Cardiologia, São Paulo 04012-909, SP, Brazil; jose.alencar@dantepazzanese.org.br; 2 Medical College of Wisconsin, Wauwatosa, WI 53226, USA; hanschelseth@gmail.com; 3 Faculdade de Medicina de São José do Rio Preto, São Paulo 15090-000, SP, Brazil; henrique.assis@edu.famerp.br; 4 Department of Emergency Medicine, University of Minnesota Hennepin Healthcare, 516 Delaware St SE, Minneapolis, MN 55455, USA

**Keywords:** Bayesian reasoning, occlusion myocardial infarction, ECG interpretation, likelihood ratios, Fagan nomogram

## Abstract

**Background**: Millimetric ST-segment elevation (STEMI) rules miss more than half of angiographic coronary occlusions. Re-casting acute infarction as Occlusion MI (OMI) versus Non-Occlusion MI (NOMI) and embedding that paradigm in Bayesian reasoning could shorten time to reperfusion while limiting unnecessary activations. **Methods**: We derived age- and sex-specific baseline prevalences of OMI from national emergency-department surveillance data and contemporary angiographic series. Pre-test probabilities were adjusted with published likelihood ratios (LRs) for chest-pain descriptors and clinical risk factors, then updated again with either (1) the stand-alone accuracy of ST-elevation or (2) the pooled accuracy of a broader OMI ECG spectrum. Two decision thresholds were prespecified: post-test probability >10% to trigger catheterization and >75% to justify fibrinolysis when angiography was unavailable. The framework was applied to five consecutive real-world cases that had elicited diagnostic disagreement in clinical practice. **Results**: The Bayesian scaffold re-classified three “NSTEMI” tracings as intermediate or high-probability OMI (post-test 27–65%) and prompted immediate reperfusion; each was confirmed as a totally occluded artery. A fourth patient with crushing pain and a normal ECG retained a 17% post-ECG probability and was later found to have an occluded circumflex. The fifth case, an apparent South-African-Flag pattern, initially rose to 75% but fell after a normal bedside echo and normal troponins. **Conclusions**: Layering pre-test context with sign-specific LRs transforms ECG interpretation from a binary rule into a transparent probability calculation. The OMI/NOMI Bayesian framework detected occult occlusions that classic STEMI criteria missed.

## 1. Introduction

The traditional STEMI/NSTEMI taxonomy, codified in international guidelines, relies on millimetric ST-segment thresholds that were never prospectively derived for sensitivity or mortality reduction. Contemporary meta-analysis shows sensitivity as low as 43.6% for STEMI criteria, leaving nearly half of occlusions untreated in time [1]. Consequently, investigators propose re-framing acute MI into Occlusion MI (OMI) versus Non-Occlusion MI (NOMI) [2,3]. Importantly, the same meta-analysis showed that when an experienced physician evaluated the complete spectrum of occlusion-specific ECG criteria simultaneously, sensitivity increased to 78.1%, while specificity was only slightly lower at 94.4%.

OMI denotes an acute culprit coronary occlusion or near-occlusion that produces insufficient antegrade/collateral flow—typically TIMI 0–1, or a flow-limiting lesion without adequate collaterals—and is expected to benefit from immediate reperfusion, irrespective of whether guideline ST-elevation thresholds are met. NOMI denotes acute MI without angiographic or imaging evidence of a culprit occlusion. Parallel to this paradigm shift, diagnostic cognition is evolving from binary rule-in/rule-out heuristics toward Bayesian updating, which explicitly combines pre-test probability with test performance to yield individualized post-test probability [4]. When applied to ECG interpretation, likelihood ratios derived from pattern-specific accuracy studies quantify how much a given tracing should sway our suspicion of ACO.

Bayesian reasoning treats diagnosis as a continuous reallocation of probability: an initial (pre-test) estimate, grounded in disease prevalence and patient context, is mathematically updated by the likelihood ratio of each new finding [5]. Fagan’s classic nomogram provides a one-stroke, bedside implementation of that calculation drawing a straight line from the pre-test probability through the LR of a test result [6]. Because LRs are multiplicative, successive observations—history, biomarkers, ECG features—can be layered in sequence, with each step refining the odds that an acute coronary occlusion is truly present [7].

In the pages that follow, we apply this framework systematically. For every vignette we begin with an age- and sex-specific anchor probability, adjust it with symptom-based probabilities, and finally incorporate the ECG-specific LR either for isolated ST-segment elevation or for the broader constellation of OMI patterns. Each intermediate odds calculation is converted back to probability with the Fagan nomogram, making the entire update transparent and reproducible. Importantly, this scaffold is didactic—an illustration of process—not a prospective application; the cases were previously published and were not managed using this framework. This manuscript presents five illustrative cases that expose pitfalls of the STEMI paradigm and demonstrate how Bayesian reasoning merges with the OMI framework to improve clinical judgment and resource stewardship.

## 2. Methods

This is a methodological demonstration, not a derivation/validation exercise. We did not train, calibrate, or internally validate a predictive model. Instead, we applied published likelihood ratios and pre-specified action thresholds to previously published, de-identified cases to illustrate workflow and decision impact.

### 2.1. Premises for Bayesian Updating

Accurate Bayesian updating requires two explicit building blocks: (1) a **data-driven pre-test probability** anchored to the patient’s age and sex, and (2) **likelihood ratios (LRs)** for bedside findings that precede the ECG. The values below—summarized in Table 1 and Table 2—provide a reproducible framework that we apply to every case in this article. Before proceeding it is worth clarifying how the update is performed. An LR cannot be multiplied directly by probability; the probability must first be converted to odds (Odds = P/[1 − P]). The pre-test odds are then multiplied by the LR to yield post-test odds, which can be reconverted to probability (P = Odds/[1 + Odds]). To keep the arithmetic transparent at the bedside, we use Fagan’s nomogram, a tool that performs these two transformations graphically without the need for calculators [6]. Readers preferring natural-frequency formats can regard the LR as the factor by which the number of “occlusion” tickets in a bag is multiplied after each new clue—exactly the same logic rendered in everyday numbers.

### 2.2. Baseline Probability of Occlusion MI Among Chest-Pain Presentations

National ED surveillance shows that ST-elevation MI (STEMI) accounts for only a handful of visits per 10,000 adults, but the rate rises steeply with age [8]. Chest pain represents ≈ 5% of all ED encounters [9]; therefore, dividing STEMI incidence by five converts the population figure into STEMI prevalence within the chest-pain cohort. Contemporary angiographic series demonstrate that roughly 30% of NSTEMI patients have a totally occluded culprit artery; adding this fraction means **Occlusion MI (OMI) ≈ 1.7 × STEMI** [10]. The resulting age-dependent baseline probabilities are presented in Table 1 and serve as the “anchor” odds before any symptoms, risk factors, troponin, or ECG data are applied. These anchors are expressed for women; the odds for men are obtained by multiplying by 1.3 at any age.

### 2.3. Pre-ECG Clinical Multipliers

Fanaroff’s systematic review and network meta-analysis quantified how individual symptoms, and historical features alter the likelihood of acute coronary syndrome (ACS) [11]. Although the meta-analysis pooled both STEMI and NSTEMI, its LRs are the best available evidence for *pre-test* modifiers and translate directly to OMI risk. We list the principal findings in Table 2. In the case discussions we multiply the anchor odds from Table 1 by every LR that applies, convert back to probability, and **only then** add the ECG-specific LR using a Fagan nomogram. We anchored pre-test estimates with Fanaroff’s pooled LRs for chest-pain descriptors; lesion-localizing symptom topography likely carries additional information, but validated, sign-specific LRs are sparse and were not applied here. The framework can incorporate such LRs as they become available.

**Table 2:** Pre-ECG likelihood ratios.

Clinical Feature	LR+ (95% CI)	LR− (95% CI)
“Typical” chest pain	1.9 (0.94–2.9)	0.52 (0.35–0.69)
Radiation to both arms	2.6 (1.8–3.7)	0.93 (0.89–0.96)
Pain identical to prior ischemia	2.2 (2.0–2.6)	0.67 (0.60–0.74)
Change in pain ≤ 24 h	2.0 (1.6–2.5)	0.84 (0.79–0.90)
Worse with exertion	1.5–1.8	0.66–0.83
Radiation to neck or jaw	1.5 (1.3–1.8)	0.91 (0.87–0.95)
Recent similar episode	1.3 (1.1–1.4)	0.80 (0.71–0.90)
Radiation to left arm	1.3 (1.2–1.4)	0.88 (0.81–0.96)
Radiation to right arm	1.3 (0.78–2.1)	0.99 (0.96–1.00)
Associated diaphoresis	1.3–1.4	0.91–0.93
Burning quality	1.0–1.4	0.97–1.00
Nausea or vomiting	0.92–1.1	0.98–1.00
Palpitations	0.71 (0.37–1.3)	1.00 (0.98–1.10)
Syncope	0.55 (0.39–0.76)	1.10 (1.10–1.10)
Pleuritic character	0.35–0.61	1.10–1.20

Legend: Pre-ECG likelihood ratios from Fanaroff et al. [11]. Ranges without parentheses indicate the span of individual-study estimates when a pooled CI was not reported.

### 2.4. ECG Evaluation

When that final ECG step is reached, two accuracy tiers are available. Using ST-segment elevation alone, sensitivity is 43.6% and specificity 96.5% (LR+ ≈ 12.5, LR− ≈ 0.59, diagnostic odds ratio—DOR 21.2) [1]. A more comprehensive reading that also recognizes hyperacute T waves, de Winter morphology [12], Aslanger pattern [13], precordial swirl [14], and related signs increases sensitivity to 78.1% with a modest drop in specificity to 94.4% (LR+ ≈ 14, LR− ≈ 0.23) [1]. We select whichever tier best fits the tracing under review and cite the corresponding LR.

### 2.5. Thresholds to Act

Classical decision theory describes two pivot points on the probability axis. The testing threshold marks the point below which neither testing nor treatment is worthwhile; the treatment threshold marks the point above which therapy begins without further confirmation. Mathematically those thresholds are given by the Pauker–Kassirer expressions, which balance the net benefit of treatment (Brx), the net harm of unnecessary treatment (Rrx), the intrinsic risk of the test (Rt), and the conditional probabilities of true- and false-positive results [15]. We set pragmatic targets that honor the qualitative direction in which the formulas push the decision lines for OMI.

- **Cath-lab activation.** In this article we treat a post-test probability **>10%** as sufficient to mobilize the catheterization team because (a) OMI carries high short-term mortality; (b) angiography both confirms and immediately treats the lesion; and (c) the procedural risk of diagnostic coronary angiography in modern practice is extremely low. It is important to emphasize that this threshold is individual, not population-level. Crossing the 10% post-test mark simply means that, given everything we know about this one patient, his or her chance of a culprit occlusion is higher than the procedural risk of a diagnostic angiogram plus the opportunity cost of mobilizing the team. It does not imply that 90% of activations would be false-positive across an entire service line.
- **Systemic thrombolysis.** Fibrinolytic therapy, in contrast, is delivered before an anatomic diagnosis and carries a non-trivial hemorrhagic risk. We therefore reserve thrombolysis for cases in which the Bayesian post-test probability **exceeds 75%**.

### 2.6. Ethical Considerations—Use of Publicly Available Material

All five clinical vignettes (ECGs, troponins, angiographic outcomes) were obtained from Dr Smith’s ECG Blog, an open-access educational site that publishes cases only after removal of direct identifiers. The framework presented in this manuscript was not applied prospectively to these patients.

## 3. Results

### 3.1. Case 1—Teenager with Classic Anterior STE

A 17-year-old male with no known personal or familial cardiac medical history presented to the emergency department (ED) with around 1–2 h of chest pain. His ED ECG shows ST elevation meeting criteria for STEMI across leads V2–V6. His anterior T waves are hyperacute. His ECG was interpreted by emergency providers as benign early repolarization.

Using the age-band anchor from Table 1, an 18–34-year-old patient with chest pain carries a baseline 0.17% probability of an occlusion MI. Multiplying by the male-sex factor (×1.3) yields 0.21%. The pain was typical—central, exertional, unrelenting—and developed within the previous 24 h; Fanaroff’s meta-analysis assigns likelihood ratios of 1.9 and 2.0 to those features, respectively. Sequentially applying those multipliers results in a pre-ECG probability of 0.8%.

**Figure 1:**
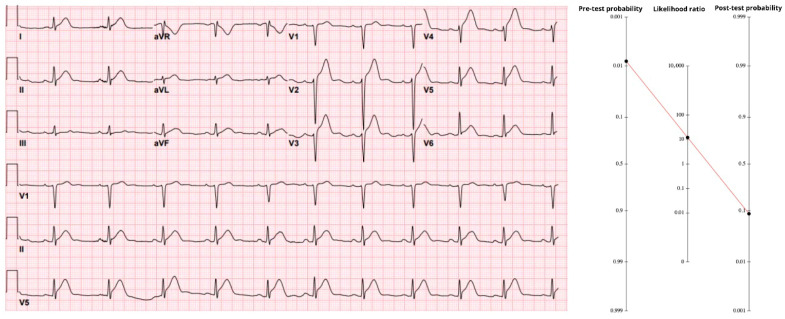
Legend: Fagan nomogram linking a 0.8% pre-test probability to a 9% post-test probability after application of an LR+ 12.5 for classic anterior STEMI. The accompanying ECG shows 4–6 mm ST-segment elevation with hyper-acute T waves in leads V2–V6, consistent with proximal LAD occlusion. https://drsmithsecgblog.com/a-teenager-with-chest-pain-troponin/ (accessed on 1 July 2025).

The initial ECG (Figure 1) showed 4–6 mm of ST-segment elevation in V2–V6 with hyperacute anterior T waves—classic evidence of proximal LAD occlusion. Using the stand-alone accuracy of anterior ST-elevation (sensitivity 43.6%, specificity 96.5%), the positive likelihood ratio is 12.5. When that value is applied to the pre-ECG probability, the result is 9.2%, giving a post-ECG probability just under 10%. In an adolescent, a 9% probability of coronary occlusion must call for attention. Notably, when the same tracing was processed through the Queen of Hearts neural network, the model returned a raw score of 1000—its maximal output. That value corresponds to 100% specificity, i.e., no false positives recorded. Treating such a result as an “infinite” LR+ would shove the post-test probability for this individual essentially to 100%.

A repeat ED ECG showed a new right bundle branch block and persistent STE. After review with cardiology, it was determined that the patient did not need emergent catheterization. He was admitted to the pediatric intensive care unit where serial troponins rose above the limit of detection and echocardiography showed an ejection fraction of 10% and left anterior descending artery (LAD) distribution hypokinesis. Coronary angiography 5 days after presentation showed total thrombotic LAD occlusion.

Therefore, the conclusion of this case highlights that clinical decisions should not be based solely on patient age. Even when the pre-test probability is estimated as low as 0.8%, application of Bayes’ Theorem via the Fagan nomogram demonstrates that the post-test probability of an acute coronary occlusion can increase to approximately 10%, and yet higher than 10% if the specificity of the ECG is extremely high. Such a finding must not be overlooked in the emergency setting, as dismissing it could result in serious patient harm or delayed treatment with potentially severe consequences.

### 3.2. Case 2—Pericarditis-like ECG

A woman in her early forties, treated for hyperlipidemia and still smoking a half-pack per day, arrived pale and “pacing in the room,” clutching her chest. The substernal discomfort radiated to both axillae and into the upper back; she could localize no positional or pleuritic component. Baseline probability for an occlusion MI in a 35-to-44-year-old chest-pain patient is 1.4%. That anchor rises with radiation to both arms (LR 2.6) and with pain that clinicians recognize as “typical” ischemic (LR 1.9). Taken together those multipliers lift the pre-ECG probability to 6.6%.

**Figure 2:**
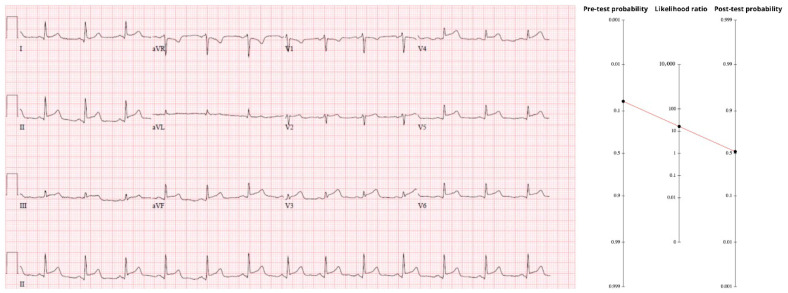
Legend: Fagan nomogram demonstrating the jump from a 6.6% pre-test probability to ≈50% once an LR+ ≈ 14 is applied for occlusion-specific findings. The ECG displays diffuse, concave ST-segment elevation without reciprocal depression, a pattern that can masquerade as pericarditis. https://drsmithsecgblog.com/you-diagnose-pericarditis-at-your-peril/ (accessed on 1 July 2025).

The initial tracing (Figure 2) showed diffuse ST-segment elevation in II, III, aVF, and V3–V6, without the expected reciprocal depression in aVL. An automated interpretation and the first reviewer called it pericarditis. Closer inspection revealed terminal QRS distortion in V3—absence of both an S-wave and a J-wave—an abnormality never seen in benign early-repolarization and highly specific for proximal LAD occlusion [16]. Because the pattern contains more than mere millimeters of STE, the comprehensive OMI criteria apply (sensitivity 78.1%, specificity 94.4%, LR+ ≈ 14). Multiplying the pre-test odds by that LR drives the post-test probability to about 50%. A one-in-two chance of an acutely occluded artery mandates immediate reperfusion. Coronary angiography was performed the morning after presentation and revealed a totally occluded mid-LAD artery.

A head-to-head, consecutive-series study that contrasts pericarditis markers—Spodick sign, diffuse STE without reciprocal change, PR depression—against unequivocal occlusion cues such as terminal QRS distortion has never been published. In that evidentiary vacuum, the calculus pivots on relative harm. Missed pericarditis rarely kills; missed LAD occlusion often does. Put bluntly, “diagnose pericarditis at your own peril.” Accepting a small excess of cath-lab activations is a trivial price for averting the catastrophic morbidity of an untreated OMI.

In a counter-factual exercise, keep the same anchor of 1.4%. A pleuritic chest pain carries a likelihood ratio of about 0.5, cutting the odds in half and dropping the pre-ECG probability to ≈0.8%. If the ECG only showed diffuse, concave ST-elevation without any occlusion-specific signs, applying the negative LR for “no OMI signs” (≈0.23) drives the post-test probability below 0.2%. At that level the Bayesian calculus strongly favors a conservative work-up for pericarditis rather than emergent reperfusion.

### 3.3. Case 3—High Pre-Test, Normal ECG

A 75-year-old hypertensive, hyperlipidemic man arrived by ambulance after ninety minutes of crushing chest pain with vomiting, unrelieved by three sub-lingual nitroglycerin tablets and 325 mg of aspirin. The baseline probability of occlusive MI for a 75-to-84-year chest-pain patient is 16%. Applying the male multiplier (×1.3) raises that to 0.20. The pain was typical and radiated posteriorly (LR+ 1.9) and was accompanied by diaphoresis and emesis (LR+ 1.3); sequential multiplication pushes the pre-ECG probability to 38%.

The first high-sensitivity troponin-T, drawn ten minutes after arrival, measured 32 ng/L. A single presentation sample above the 99th percentile detects STEMI with >90% sensitivity but only 35% specificity, yielding an LR+ of 1.4 and an LR− of ~0.28 [17]. Applying that modest LR+ pushes the probability from 38% to 46%, a clinically meaningful increase but far from definitive.

**Figure 3:**
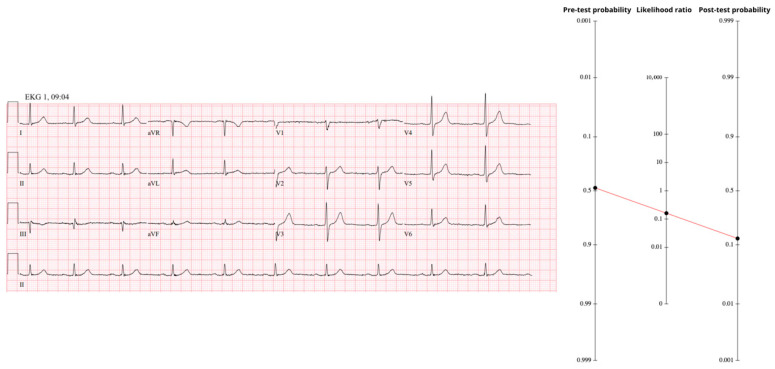
Legend: Fagan nomogram showing how a normal ECG (LR− ≈ 0.23) lowers the probability from 46% to 17%. The ECG is normal sinus rhythm with no ischemic changes—emphasizing that a “clean” tracing does not rule out an occluded artery. https://drsmithsecgblog.com/chest-pain-and-completely-normal-ekg/ (accessed on 1 July 2025).

The ECG recorded at the same moment was completely normal (Figure 3); absence of every occlusion-specific sign invokes the negative likelihood ratio for the full OMI spectrum (LR− ≈ 0.23). Using Fagan’s nomogram results in a post-ECG probability of 17%, still above our 10% catheterization trigger despite the “reassuring’’ tracing.

Serial troponins climbed to 48 ng/L and 80 ng/L while the ECG remained unchanged. Serial troponin elevation carries 100% sensitivity and 83.7% specificity for OMI, generating an LR+ of ≈6.1 [18]. This shifts the probability to ≈55% of having an occluded artery, beyond threshold for invasive confirmation. Cardiology deferred invasive study, ordering CT coronary angiography instead; the scan, completed at 14:22, showed a totally occluded mid-circumflex artery with transmural ischemia of the basal lateral wall. Invasive angiography at 16:04 confirmed plaque rupture and thrombotic occlusion; aspiration and stenting restored flow. A next-day echocardiogram revealed basal-to-mid-lateral hypokinesis with an ejection fraction of 55–60%, and the patient was discharged two days later with the label “NSTEMI.” Therefore, not using all available information, or not recognizing it, or not using it appropriately to construct a Bayesian probability, resulted in significant loss of myocardium.

### 3.4. Case 4—Intermediate Pre-Test, Precordial Swirl Pattern

A previously healthy 40-year-old woman was cooking dinner when she felt a sudden, tight retro-sternal pressure. She swallowed an oxycodone tablet, but the pain persisted, and EMS was called. On arrival she was diaphoretic yet hemodynamically stable. Because she is a 40-year-old female with new chest pain, the age-band anchor from Table 1 is 1.4%. Applying a single “typical ischemic pain” multiplier (LR 1.9) lifts the pre-ECG probability to 2.6%.

**Figure 4:**
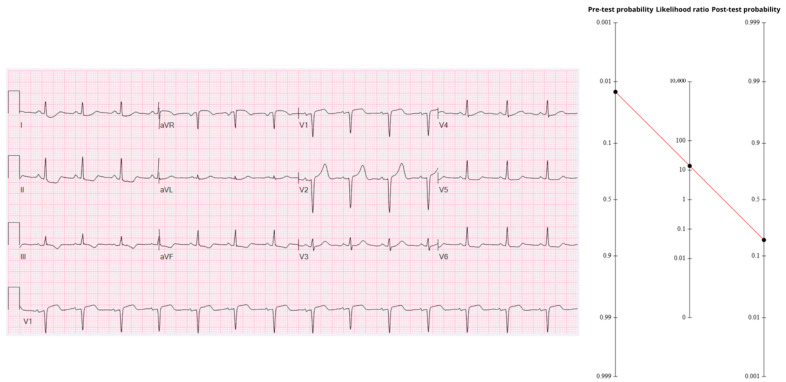
Legend: Fagan nomogram illustrating the rise from a 2.6% pre-test probability to ≈27% after applying an aggregate LR+ ≈ 14 for the general OMI findings. The ECG shows 1–2 mm ST elevation in V1–V2, a hyper-acute T wave in V2, and reciprocal ST depression in V4–V6—the precordial swirl sign of proximal LAD occlusion. https://drsmithsecgblog.com/the-dye-dont-lie-except-when-it-does/ (accessed on 1 July 2025).

The first ECG (Figure 4) showed 1–2 mm ST-elevation in V1–V2, hyper-acute T-waves in V2, and reciprocal ST-depression in V4–V6—together forming the precordial-swirl pattern classically associated with a proximal LAD occlusion [14]. Using the aggregate OMI accuracy figures (Sn 78.1%, Sp 94.4%, LR+ ≈ 13.95), the 2.6% pre-ECG probability converts to a probability of approximately 27%.

An initial high-sensitivity troponin-I returned 10 ng/L (below the female 99th-percentile); the LR- for a negative presentation sample (~0.28) pushed probability downward to 9.4%. Two hours later, troponin levels went to 8512 ng/L, instantly ruling-in myocardial necrosis. Bedside echo showed mid-to-apical anterior hypokinesis, and the patient was rushed to angiography.

Conventional cine views appeared deceptively normal—so normal, in fact, that many operators might have closed the case as “non-obstructive CAD.” Intravascular ultrasound, however, unmasked a ruptured plaque in the proximal LAD extending into the distal left main, exactly matching the swirl prediction. For technical reasons, it was not stented.

Troponin peaked at 36,029 ng/L and the post-PCI ECG showed classic LAD reperfusion T-wave inversion. Recurrent pain a few hours later prompted repeat angiography, and this time a prophylactic stent was ultimately placed across the culprit site.

Every datapoint—young age for ACS, a single “negative” troponin, a subtle finding on ECG—conspired to lure clinicians into premature closure. Yet a 27% post-ECG probability still means a high individual risk and when the downside is transmural infarction, there is nothing like “low risk”, especially in the LAD distribution [19]. Serial evaluation is the antidote: repeat tracings after nitroglycerin, second-look echoes, and a second troponin turned hours into minutes all enlarging the diagnostic aperture. In other words, Bayesian updating is not a one-off calculation but a live, iterative loop.

### 3.5. Case 5—False-Positive OMI Finding (South African Flag Sign)

A 58-year-old man summoned EMS for stabbing, left-sided chest pain radiating to the shoulder and accompanied by nausea and diaphoresis. In the 55-to-64-year-old male chest-pain stratum, the baseline OMI prevalence is 9%. “Typical” anginal character raises those odds by a factor of 1.9; radiation to the left arm adds 1.3; and autonomic symptoms contribute little (LR ≈ 1). Pre-ECG probability therefore climbs to 19.6%.

**Figure 5:**
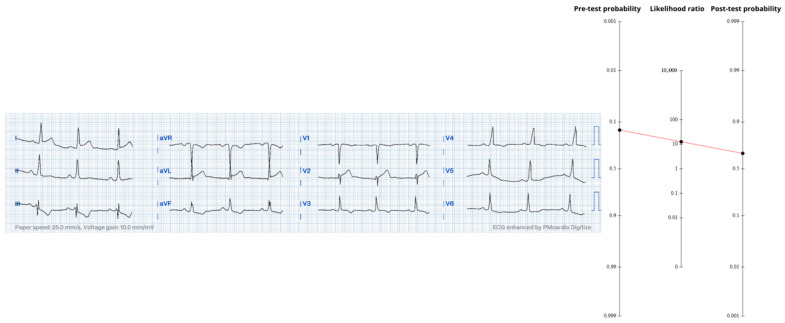
Legend: Fagan nomogram converting a 19.6% pre-test probability to 75% post-test probability using an LR+ ≈ 12.5 for the South-African-Flag sign. The ECG reveals ST elevation in leads I, aVL and V2 with reciprocal depression in III, forming the South-African-Flag configuration. https://drsmithsecgblog.com/cath-lab-occupied-which-patient-should/ (accessed on 1 July 2025).

The field ECG (Figure 5) displayed upward ST-shift in I-aVL with reciprocal ST-depression in III and dynamic ST-T morphology in V2—an appearance touted as the South-African-Flag sign of mid-anterior OMI. The presence of millimetric STEMI has LR⁺ of ≈12.5. Fagan’s nomogram raises post-ECG probability to 75%—well above the 10% cath-activation trigger and even flirting with the 75% lytic threshold.

It is important to emphasize that the authors were not unanimous on that interpretation. One of us saw subtle terminal QRS-distortion in V2 and judged the tracing “highly suspicious,” while another, supported by the Queen-of-Hearts algorithm (raw output = 0.03, i.e., “not OMI”), considered the pattern far more likely to be non-specific ST elevation.

Bedside echocardiography showed a strongly contracting left ventricle with no regional wall-motion abnormality, a finding that sharply lowers the likelihood of an occlusive infarct. Two hours later the first and second high-sensitivity troponin-T results were still at the low-normal range, further downgrading the odds. By the time these bedside tests were in hand, the Bayesian curve had changed from “almost certainly OMI” on the first ECG to “very unlikely.” Cardiology proceeded to the lab emergently upon arrival to the ED: the coronaries were normal except for a smooth, 30% irregularity in the mid-LAD, with no culprit thrombus or plaque rupture.

Had this patient been fibrinolyzed in the pre-hospital phase—a defensible choice, at least for one of us, given the initial appearance—he would have received medication for a disease he never had. That tension defines modern chest-pain care: there is no shame in the cautious false positive if the alternative is a missed occlusion, yet every escalation carries its own price. As Osler reminded us, medicine is “a science of uncertainty and an art of probability.” The maturity lies in knowing when to keep updating the probability—and when to stop.

## 4. Discussion

### 4.1. Bayesian Reasoning: From Theory to Bedside

The relationship between accurate diagnosis and appropriate treatment is fundamental to the principle of medical practice. When the diagnosis is correct, there is a significantly higher probability that subsequent therapeutic decisions will also be appropriate [20]. Establishing a diagnosis is, therefore, a critical step in clinal decision-making. Clinicians, when evaluating a patient, must consider a multitude of clinical indicators, often under conditions of uncertainty and probabilistic reasoning [5]. In most of the scientific literature, the performance of diagnostic tests is primarily described in terms of sensitivity and specificity. Sensitivity refers to the probability that tests will correctly identify a patient with the disease (true positive), whereas specificity refers to the probability that tests will correctly identify a patient without the disease (true negative) [21]. However, in real-time clinical scenarios, the clinician does not have certainty regarding the patient’s disease status. Such sensitivity and specificity are considered post hoc measures, calculated retrospectively based on known outcomes [22].

The incorporation of Bayesian reasoning into clinical practice has enhanced diagnostic precision by providing a structured framework for integrating prior knowledge with new clinical data. A particularly robust tool within this framework is the use of LRs, both positive (LR+) and negative (LR−). The LR represents the odds that a given test result would be expected in a patient with the disease compared to a patient without it. It quantifies how many times more likely it is that the patient has, or does not have, the disease. An LR+ greater than 1 suggests that the patient is more likely to have the disease based on the diagnostic test result, whereas an LR− less than 1 indicates that the patient is less likely to have the disease. Therefore, it can be summarized that the further the likelihood deviates from 1, the greater its impact on increasing or decreasing the ratio that the patient has [23]. The diagnostic odds ratio combines both LR+ and LR− into one number, dividing one by the other. For instance, if a positive test increases the likelihood by 10×, and a negative test decreases the odds of disease by 10× (to 0.10, or 1/10th), then the “diagnostic odds ratio” for such a test is very high, at 10 divided by 0.10 = 100. This indicates a very valuable test.

The practical application of Bayes’ Theorem in clinical settings is exemplified by the Fagan Nomogram, a graphical tool designed to visually integrate the pre-test probability with the likelihood ratio in order to derive a post-test probability [6]. This method allows clinicians to incorporate evidence-based data in real-time decision-making, making it a valuable resource for probabilistic reasoning in medical diagnostics. In general terms, a nomogram consists of three aligned scales, each representing a distinct variable. The primary objective is to draw a straight line connecting two known values, with the third scale providing the resulting value derived from this alignment. In the context of clinical diagnostics, the first scale represents the pre-test probability, the second corresponds to the likelihood ratio, and the intersection of these two yields the post-test probability, that is the updated probability that the patient has the disease based on findings from the physical examination or a diagnostic test [24,25].

### 4.2. OMI/NOMI: A Paradigm Aligned with Bayes

The STEMI/NSTEMI paradigm is the mainstream diagnosis since the beginning of the fibrinolytic era: one surrogate finding on a single test (millimetric ST-elevation on an ECG) became the gatekeeper to reperfusion therapy. That binary shortcut simplifies triage but silently embeds two false premises: (a) absence of ST-elevation approximates absence of occlusion, and (b) the ECG interpreter, not the angiogram, is the final arbiter of pathology. Pooled accuracy data now refute the first premise—classic STEMI criteria capture barely 44% of angiographically proven acute occlusions—while showing that broader occlusion-specific signs (hyper-acute T waves, de Winter, Aslanger, swirl, terminal QRS-distortion, etc.) boost sensitivity to 78% with only a modest fall in specificity to 94.4% [1]. The second premise falls once we rename the disease: Occlusion Myocardial Infarction states explicitly that the gold standard is a catheter tip in the coronary lumen, not a ruler on an ECG. The vocabulary forces clinicians to ask, “Is the artery open?” rather than, “Does the ST-segment meet a guideline threshold?”—a shift from pattern matching to pathophysiology. In our series every true-positive OMI would have been missed or delayed under a STEMI-only screen, yet every false positive reminded us that ECG is still a surrogate. OMI/NOMI paradigm therefore invites Bayesian thinking by construction: it treats each ECG sign as one likelihood ratio among many on the path toward the angiographic truth.

Operationally, OMI/NOMI tends to bring more STEMI-negative occlusions (often labeled NSTEMI) to early angiography. This framework should never be used to justify deferring invasive evaluation when clinical risk warrants it; its purpose is to surface occlusions that millimetric criteria miss.

### 4.3. OMI/NOMI: A Paradigm That Is Aware of Its Own Biases

Abandoning the STEMI label does not grant omniscience; it merely puts error where it belongs—on a probability scale instead of behind a binary curtain. The word “occlusion” warns us that even a pristine tracing or a non-diagnostic troponin can coexist with an occluded artery (Case 3), while the word “non-occlusion” concedes that an ECG that is apparently diagnostic by millimeter criteria can still be a false positive by expert or AI interpretation (Case 5). By acknowledging up front that all bedside signs are fallible surrogates, the paradigm counters persistent biases present in EDs: anchoring and giving premature closure on a single negative ECG. Physicians must, then, perform iterative verification—serial tracings, second-look echoes, repeat biomarkers—and an intellectual humility that keeps the cath-lab threshold low but not indiscriminate. In short, OMI/NOMI does not pretend to erase diagnostic error; it keeps that error visible, measurable, and, most importantly, refreshable as new data arrive.

## 5. Limitations

The Bayesian framework we present stands on numerical premises—anchor prevalences, likelihood ratios, and action thresholds—that some readers will judge too liberal or too conservative. That divergence is expected. Probability is not a verdict but a starter, inviting others to exchange their own priors and regenerate the post-test curves. Our 10% catheterization and 75% fibrinolysis cut-points, for example, make sense in systems where door-to-wire times are short and neurologic support is present and strong; in centers with longer delays or higher hemorrhagic risk, those break-points should slide to the right. It is also deliberately didactic. These thresholds provide clear landmarks for teaching diagnostic reasoning; they do not imply that the authors (or any thoughtful clinician) calculate a precise post-test probability at the bedside and then flip a reperfusion switch whenever the dial crosses a bright line. This five-case series is didactic and not powered for inference; it cannot support calibration, inter-rater reliability estimates, or external validation. Our aim was to demonstrate how published LRs and prespecified thresholds can be used prospectively, not to derive new thresholds or a multivariable prediction model.

In some cases above, we applied the aggregate accuracy of the full OMI spectrum (Sn ≈ 78%, Sp ≈ 94%) instead of the accuracy for the single ECG sign actually present (e.g., precordial swirl, Aslanger, isolated ST-elevation). We did so because many newly described patterns have never been validated in isolation; their stand-alone sensitivities and specificities simply do not exist. Pooling them under the “any OMI sign” umbrella was therefore a pragmatic stop-gap, not an ideal solution. As dedicated accuracy studies emerge for each pattern, future iterations of this framework should substitute those sign-specific LRs.

Probability-based activation will inevitably shift some activations earlier and may increase false positives in certain systems. These resource and cost trade-offs are context-specific and must be weighed against the morbidity of delayed reperfusion; formal cost and resource-use analyses were beyond scope. From a medico-legal standpoint, we recommend documenting the estimated pre- and post-test probabilities and the rationale for acting at a given threshold to make the decision process explicit and auditable.

In applying the Fagan nomogram, we rounded intermediate and final probabilities to simplify presentation and align with clinical utility. These rounding decisions, while minor, may slightly alter perceived risk, particularly near decision thresholds (e.g., 10% for catheterization). Such approximations reflect the practical constraints of nomogram-based calculations and clinical reporting, where precision beyond one decimal place rarely impacts decision-making. However, in high-stakes contexts, these differences could influence borderline decisions, and clinicians should be aware that exact calculations may yield slightly higher or lower probabilities.

Clinical reasoning extends beyond tabulated LRs. Much of the “experienced gestalt” that shapes a clinician’s prior—an anxious spouse’s glance, a tremor in the patient’s voice, the incongruent calm of someone who claims excruciating pain—has never been captured in chest-pain cohorts. Bayesian interpretation cannot yet price these subtleties. On the other hand, these subjective aspects of clinical reasoning are very difficult to subject to scientific scrutiny. The same happens with ECG interpretation: a memory of a prior case or a pattern that “just feels wrong” because it violates a personal mental library of tracings inevitably shifts each clinician’s post-test estimate in slightly different directions. Additionally, we anchored clinical priors with Fanaroff’s meta-analysis because it provides the most comprehensive pooled LRs for chest-pain descriptors, but it does not exhaust all potentially informative features. Symptom topography and other clinical signals may further refine priors. The same applies to comorbidity context: this data can and must be added to Bayesian framework if robust.

ECG expertise is very variable. For some of the ECGs presented, one author (SWS) would apply a much higher specificity and thus much higher LR+. The Queen of Hearts AI Model only diagnoses OMI with a specificity of 98% when the raw output value is 0.50, and with higher specificity when that number is closer to 1.0. The overall sensitivity is 80% at an overall specificity of 98%. This would give an +LR of 40.

For didactic clarity we treated the various clinical and ECG findings as statistically independent and multiplied their likelihood ratios in sequence. In reality, LRs behave like odds ratios: when two predictors are colinear—say, hyper-acute T waves and terminal QRS-distortion in the same anterior lead—a multivariable model would adjust their weights, often shrinking one toward the null. Our serial-multiplication approach almost certainly overestimates (or, less often, underestimates) the true post-test probability in such situations [26]. Recognizing this, we opted to preserve the step-wise calculation because it lays bare the Bayesian logic; the price is a loss of mathematical precision that only large, multivariable datasets will be able to correct.

Lastly, we recognize that approximately one-third of biomarker-defined myocardial infarctions present without chest pain [27]. In the described framework the absence of characteristic symptoms would bring negative LRs that push the post-test probability further downward—but never to zero.

## 6. Conclusions

Chest pain is an archetypal probabilistic complaint: common, diverse, and potentially lethal. A diagnostic framework that treats every new data—age, character of pain, biomarker, lead-specific ECG nuance—as a likelihood ratio to be multiplied rather than a yes/no trigger is therefore indispensable. Bayesian updating supplies that framework, allowing clinicians to slide smoothly along the probability spectrum instead of stumbling over binary cut-offs. When paired with the OMI/NOMI paradigm, the approach replaces the question, “Does the tracing meet STEMI criteria?” with the better question, “How much did this finding move my estimate that an artery is closed?” Our five cases show the dividends of that shift: hidden occlusions surfaced, premature closure was averted, and false positives were explained—all through transparent recalculation rather than heroic intuition.

Implementing this mindset need not await new technology or guidelines; a pocket nomogram, serial ECGs, and a habit of asking “What are the odds now?” are enough to begin. As evidence accumulates and tools such as machine-learning ECG interpreters mature, the Bayesian scaffolding will only strengthen. Until then, integrating pre-test context with test performance remains the most reliable way to honor the first duty in chest-pain care: reperfuse the myocardium that can still be saved, and spare the patient from needless harm.

## Figures and Tables

**Table 1:** Baseline probability of Occlusion MI.

Age (yr)	STEMI Visits per 10,000 ED Encounters	STEMI Prevalence Among Chest-Pain Visits ^†^	Baseline P(OMI) ^‡^
18–34	0.5	0.10%	0.17%
35–44	4.0	0.82%	1.4%
45–54	11.7	2.34%	4.0%
55–64	20.8	4.16%	7.1%
65–74	31.3	6.26%	10.6%
75–84	47.2	9.44%	16.0%
≥85	83.4	16.68%	25% ^§^

Legend: Baseline probability of Occlusion MI in ED chest-pain visits, by age band *. * Apply a 1.3× odds multiplier for male sex at any age. ^†^ Chest-pain complaints constitute ≈ 5% of all ED visits. ^‡^ OMI = STEMI + occlusive-NSTEMI; ≈1.7 × STEMI prevalence. ^§^ Rounded down to avoid overestimation in very frail, multi-morbid populations.

## Data Availability

No new data were created or analyzed in this study.
